# Functional analysis of non-hotspot AKT1 mutants found in human breast cancers identifies novel driver mutations: implications for personalized medicine

**DOI:** 10.18632/oncotarget.755

**Published:** 2012-11-29

**Authors:** Kyung H. Yi, Jossette Axtmayer, John P. Gustin, Anandita Rajpurohit, Josh Lauring

**Affiliations:** ^1^ The Sidney Kimmel Comprehensive Cancer Center, The Johns Hopkins University School of Medicine, Baltimore, Maryland, USA; ^2^ Ponce School of Medicine and Health Sciences, Ponce, Puerto Rico

**Keywords:** AKT1, mutation, personalized, cancer, therapy

## Abstract

The phosphatidylinositol 3-kinase (PI3-kinase)-Akt-mTOR pathway is mutated at high frequency in human breast cancer, and this pathway is the focus of active drug discovery and clinical investigation. Trials of personalized cancer therapy seek to leverage knowledge of cancer gene mutations by using mutations to guide the choice of targeted therapies. At the same time, cancer genome sequencing studies are identifying low frequency variants of unknown significance in known cancer genes, as well as genes of unknown function. We have performed functional analysis of six non-hotspot AKT1 pleckstrin homology domain mutants identified in recent large-scale breast cancer sequencing studies. Three of these mutants cause constitutive activation of Akt1 in the absence of growth factors, leading to phosphorylation of downstream target proteins. Like the hotspot E17K mutation, these mutants confer constitutive membrane localization of Akt1. Finally, the same three mutants showed oncogenic activity in a cellular transformation assay. The other three mutants were inactive in all assays. These findings validate novel driver mutations in AKT1, and extend the number and type of mutations that activate the PI3-kinase pathway in human breast cancers.

## INTRODUCTION

The advent of rapid and cost-effective methods for somatic mutation analysis of cancer has brought the goal of personalized cancer therapy closer to reality. As the mutational landscapes of common tumor types are becoming clear, a number of clinical trials either completed or in progress are testing the feasibility and in some cases the efficacy of using tumor mutation data to guide selection of targeted therapy[1,2]. In some cases researchers have performed focused sequencing or mass spectrometric assays directed at known hotspot mutation sites, such as *BRAF* codon 600, *KRAS* codons 12 and 13, *PIK3CA* codons 542, 545, and 1047, and *AKT1* codon 17. However, as more large-scale genome or exome sequencing studies and more resequencing studies of known cancer genes are performed, novel variants of unknown significance are being discovered. In the cases of *PIK3CA*, *KRAS*, *NRAS*, and *ERBB2* low frequency non-hotspot mutations have been shown to have transforming activity using functional assays[3-5].

Mutational activation of the PI3-kinase-Akt-mTOR pathway is the most frequent oncogenic event in breast cancer, with a particular predilection for the hormone receptor-positive subtype of disease. The mTOR inhibitor everolimus was recently approved by the Food and Drug Administration for treatment of aromatase inhibitor-resistant estrogen receptor positive breast cancer, and a number of PI3-kinase or Akt inhibitors are under active clinical investigation in breast cancer.

The hotspot *AKT1* E17K mutation occurs in approximately 3% of primary breast cancers, exclusively in the estrogen receptor positive subtype[6-8]. The mutation occurs in the pleckstrin homology (PH) domain of Akt1 and confers constitutive plasma membrane localization in the absence of growth factor stimulation, leading to increased Akt1 activation and phosphorylation of downstream target proteins[6,9]. Recent large-scale breast cancer sequencing studies have identified other somatic sequence variants in the PH domain of *AKT1*[8,10-13]. We sought to determine whether these novel *AKT1* variants are bona-fide activating mutations. We show that several, but not all of the reported variants, confer constitutive membrane localization and activation of Akt1. These findings have implications for the evaluation of the cancer-associated somatic mutations and the implementation of personalized medicine protocols for cancer therapy.

## RESULTS

**Figure 1:**
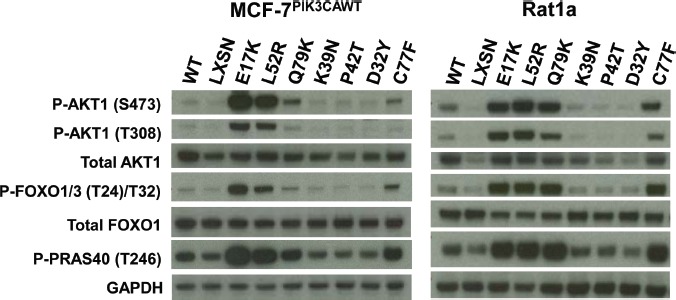
Non-hotspot AKT1 PH domain mutants L52R, C77F, and Q79K increase Akt1 activation and downstream signaling Western blot analysis of extracts from serum-starved MCF-7^PIK3CAWT^ (left panel) and Rat1a (right panel) cells stably infected with retroviruses expressing wild type human AKT1, AKT1 PH domain mutants, or no insert (LXSN).

We chose for study six *AKT1* variants reported in recent breast cancer sequencing studies: D32Y, K39N, P42T, L52R, C77F, and Q79K. L52R appears to be a recurrent mutation, as it has been identified in five independent studies to date, including one reported case of colorectal cancer[8,11-14]. K39N was identified twice in a single study, which was also the source for D32Y and P42T[12]. C77F and Q79K have been reported as single instances in different studies[10,13]. We expressed all of these variants, as well as wild type human Akt1 and the E17K mutant, in a novel derivative of the human breast cancer cell line MCF-7 created in our laboratory. MCF-7 cells have the PIK3CA helical domain mutation E545K. We used somatic cell gene targeting to replace the mutant alleles with wild type sequence. The resulting cell line, MCF-7^PIK3CAWT^ shows a drastic reduction in basal Akt activation and phosphorylation of downstream Akt targets, such as FOXO1/3 and PRAS40 compared to parental MCF-7 cells (manuscript in preparation). When expressed in MCF-7^PIK3CAWT^ the E17K mutant led to increased activation of Akt under low serum conditions, as measured by phosphorylation of serine 473 and threonine 308, when compared to empty vector control (Figure 1 and Supplemental Figure 1A). Interestingly, the over expression of wild type Akt1 also conferred some increase in activation. The novel PH-domain mutants L52R, C77F, and Q79K substantially increased Akt phosphorylation, while D32Y, K39N, and P42T did not activate Akt more than wild type. Expression of the Akt1 transgenes was confirmed by immunoblotting for total Akt1. Increased activation of Akt1 by E17K, L52R, C77F, and Q79K mutants led, as expected, to increased phosphorylation of Akt target proteins including FOXO1/3 and PRAS40. Similar results were observed in Rat1a cells transduced with the Akt1 variants (Figure 1). Although there is some variability in the expression levels of the transgenes by western blotting for Akt1, we confirmed that all of the transgenes were expressed within a two-fold range by qPCR, and we also evaluated the activity of single cell clones expressing each mutation (Supplemental Figures 1B and 2).

**Figure 2:**
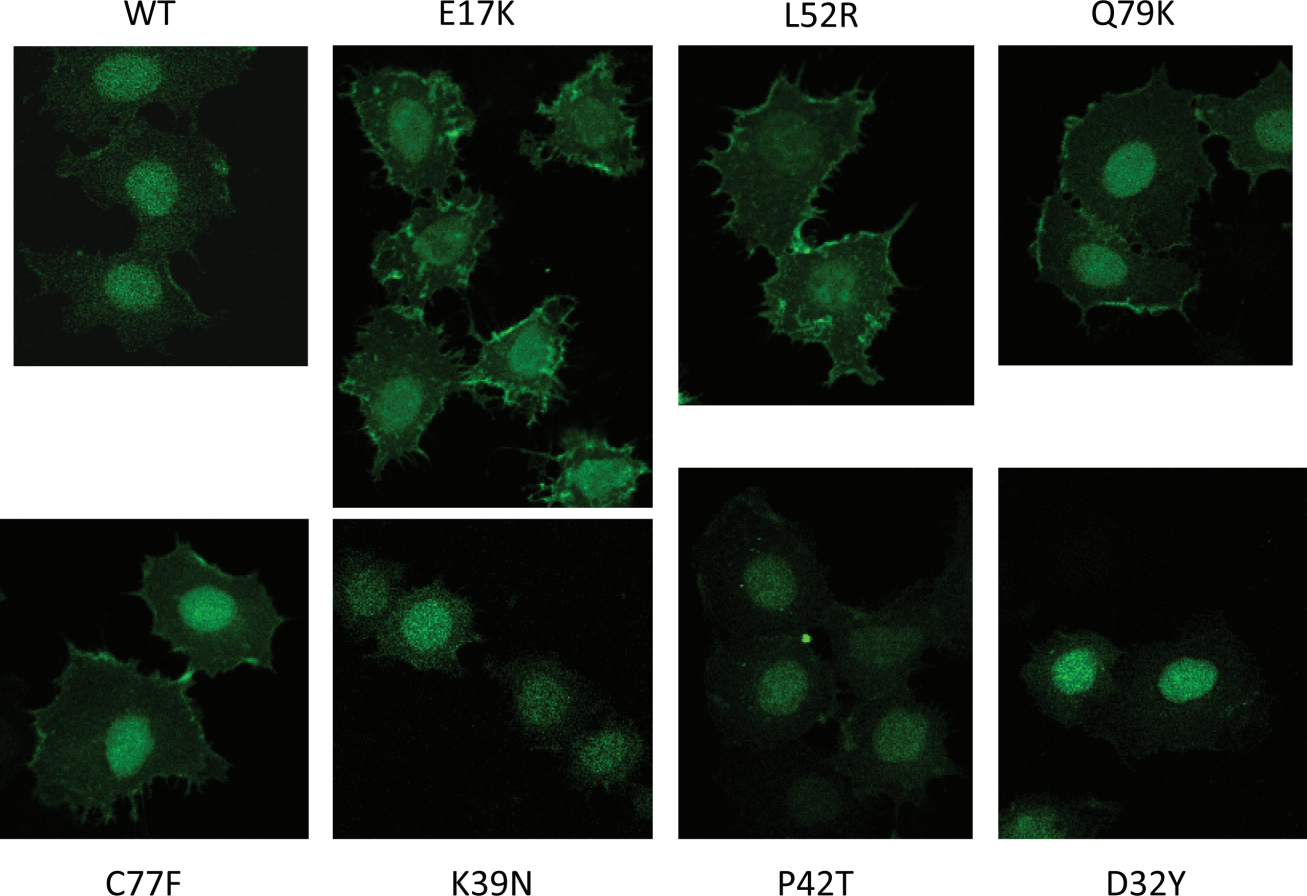
Confocal microscopy demonstrates constitutive membrane localization of Akt1 PH domain-EGFP fusion proteins under serum starved conditions in E17K, L52R, C77F, and Q79K mutants

The E17K mutation constitutively activates Akt1 by altering the kinetics and specificity of phospholipid binding, increasing the binding to PIP2, the more abundant plasma membrane phospholipid in the absence of PI3-kinase activation[9]. In order to determine whether these novel PH domain mutants similarly conferred constitutive membrane targeting of Akt1, we generated expression constructs fusing the PH domains of wild type and mutant Akt1 proteins with enhanced green fluorescent protein (EGFP). This system has been widely used to study the localization and activation of Akt, and the E17K mutant was shown to localize constitutively to the plasma membrane with this technique[6]. The AKT1-PH-EGFP fusion constructs were transfected into MCF-7^PIK3CAWT^ cells, which were serum-starved and then imaged with a confocal microscope. As previously reported the E17K PH domain confers predominant membrane localization in the absence of growth factors (Figure 2). The mutants that activate Akt-dependent signaling, L52R, C77F, and Q79K, also localize to the plasma membrane. We did not observe significant membrane localization of wild type, D32Y, K39N, or P42T PH domain fusion proteins.

**Figure 3:**
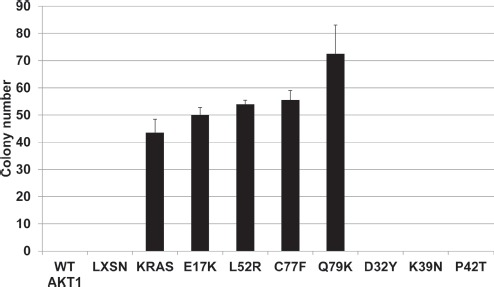
Novel AKT1 mutants L52R, C77F, and Q79K transform Rat1a fibroblasts in a soft agar colony formation assay Bars represent mean and standard deviation.

Although *AKT1* is a well characterized oncogene, we sought to demonstrate a functional effect of the mutations on cellular transformation. Wild type and mutant Akt1 proteins, along with empty vector and a Kras G12V mutant positive control were stably expressed in Rat1a cells to assess effects on soft agar colony formation. All of the Akt1 mutants that were active in signal transduction and membrane localization--E17K, L52R, C77F, and Q79K--stimulated colony formation (Figure 3). Despite the increased Akt pathway signaling observed with over expression of wild type Akt1, we did not observe an effect on colony formation, suggesting that this assay is more stringent for Akt1's oncogenic activity or that wild type Akt1 may not sufficiently phosphorylate other proteins critical for anchorage-independent growth. No colonies were observed with over expression of the three other mutants.

## DISCUSSION

We have performed biochemical and functional characterization of six novel *AKT1* pleckstrin homology domain mutants that have been identified predominantly in human breast cancers. In addition to the previously characterized E17K hotspot mutant, three other mutants were found to be activating, oncogenic mutations: L52R, C77F, and Q79K. These three mutations led to constitutive localization of the Akt1 PH domain-EGFP fusion protein to the plasma membrane in the absence of growth factors. Increased constitutive Akt activation by phosphorylation was demonstrated by western blotting, and these Akt1 mutants caused increased phosphorylation of canonical Akt target proteins, such as PRAS40 and FOXO proteins, under minimal serum conditions. Finally, these novel Akt1 mutants behaved as classical oncogenes in a transformation assay, causing Rat1a cells to form colonies in soft agar.

Structural studies have suggested that the glutamate to lysine change at amino acid 17 directly alters the lipid binding pocket of the Akt1 PH domain[6]. It is not yet clear how these novel mutations lead to constitutive membrane targeting. They could, like E17K, cause structural changes that alter the kinetics and selectivity of phosphoinositide binding, but we cannot presently exclude the possibility that they lead to increased membrane localization through a distinct mechanism. While this manuscript was under review, Parikh et al published an analysis of a number of *AKT1* mutants, including six of the seven mutants in this study[15]. In agreement with our findings they also showed biochemical activation and oncogenic activity of the L52R and Q79K mutants. The C77F mutant was not studied in their paper, however. Parikh et al [15] concluded that Akt PH domain mutants function primarily by disrupting inhibitory interactions between the PH domain and kinase domain. They showed that the E17K and Q79K mutants localized constitutively to the plasma membrane, although, in contrast to our findings, they did not observe membrane localization of the L52R mutant. We cannot explain the discrepancy between our results for this mutant.

Three other mutants, D32Y, K39N, and P42T, did not activate Akt in any of the biochemical or cellular assays. Although it is difficult to completely exclude some enhanced activity of these mutants, in the aggregate the lack of signaling over that induced by wild type Akt1, lack of membrane localization, and lack of activity in a functional transformation assay argue against D32Y, K39N, and P42T being activating mutations. In support of our results, Parikh et al [15] also found no evidence of increased Akt activity with overexpression of these same three mutants. Of note, detailed methods were not reported for how somatic sequence alterations were validated in the study that originally reported these three mutations[12]. It is possible that they represent sequencing artifacts or simply non-functional passenger mutations. This finding illustrates several important issues arising in the clinical use of genomic data for personalizing cancer therapy. First, sequencing may identify artifactual or passenger variants of no significance in known cancer genes. Use of such mutations to select therapy might result in therapeutic failure. Second, significant variants in known cancer genes may be ignored because they occur with lower frequency in non-hotspot locations. Others have shown that non-hotspot *KRAS* and *NRAS* mutations occurring in leukemias and rare *ERBB2* mutations in lung cancer are functionally transforming[4,5]. Indeed, two of the *AKT1* variants shown to be oncogenic in the current study, C77F and Q79K, have thus far been identified only once each in sequencing studies. The use of focused mutation detection technologies, such as primer extension mass spectrometric assays or sequencing only single exons (for example, *AKT1* exon 3, which does not contain C77 or Q79), will miss less common variants and potentially exclude patients from selection for targeted therapies. Some of these issues may be resolved with lower cost sequencing and improvements in sample processing or sequencing technology that reduce artifacts and allow for less-focused, unbiased interrogation of the somatic mutation landscape of patients’ tumors. Until then, the gold standard must remain functional validation of variants of uncertain clinical significance.

## MATERIALS AND METHODS

### Cell Lines

MCF-7^PIK3CAWT^ cells were derived from MCF-7 cells using gene targeting as described, except homology arms contained wild type sequence from *PIK3CA* exon 9[16]. Details of their characterization will be published elsewhere (manuscript in preparation). Compared to parental MCF-7 cells, the MCF-7^PIK3CAWT^ cells exhibit greatly reduced Akt activation and Akt-dependent signaling. The cells were cultured in DMEM (Mediatech) with 5% FBS (Hyclone) and penicillin-streptomycin (Invitrogen). Rat1a cells were obtained from Linda Smith-Resar (Johns Hopkins University) and cultured in DMEM with 10% FBS and penicillin-streptomycin. All cells were cultured at 37°C at 5% CO_2_.

### AKT1 expression constructs

Wild type and mutant versions of human *AKT1* coding sequence were cloned by PCR either directly from human cDNA or using PCR mutagenesis to introduce the various *AKT1* mutations. Primer sequences are available on request. cDNA inserts containing a Kozak consensus sequence and stop codon were cloned into the EcoRI and XhoI sites of the LXSN retroviral vector (Clontech). All sequences were verified by sequencing both strands at the Johns Hopkins Synthesis and Sequencing Facility. Constructs were packaged into VSV-G envelope pseudotyped retroviruses by transiently transfecting 293T cells using FuGENE 6 (Promega). Stable pools of cells were selected and maintained in G418 (Mediatech).

### Quantitative RT-PCR

Total RNA was prepared from cells using RNeasy kit (Qiagen) with on-column DNAase I digestion. cDNA was synthesized with a First-Strand cDNA Synthesis kit (GE Biosciences). PCR amplification was done using the MyiQ system (BioRad) with Platinum Taq polymerase and SYBR green dye (Invitrogen). Primer sequences were 5'-GCTATTGTGAAGGAGGGTTG-3' and 5'-CGTCTTCATCAGCTGGCACT for human AKT1 and 5'-CACCACTACGTGGAACGACC-3' and 5'-AATGTAGCCTGGTCGGAGC for BAG3 and 5'- GAGACCCACCTGGACATGCT-3' and 5'-ACATTGTCCTGAGGATCTCGG-3'for *ERBB2*, used as a rat reference genes. Human *AKT1* cycle number was normalized to the average of the rat *BAG3* and *ERBB2* cycle numbers using the ΔCt method.

### Immunoblotting

Whole-cell protein extracts prepared in Laemmli sample buffer were resolved by SDS-PAGE using 4-12% NuPage gels (Invitrogen), transferred to Invitrolon polyvinylidene difluoride membranes (Invitrogen), and probed with primary and horseradish peroxidase–conjugated secondary antibodies. The primary antibodies used in this study are phospho-FOXO1 (T24)/FOXO3a (T32) (Cell Signaling Technology (CST) #9464), FOXO1 (CST #2880), phospho-PRAS40 (T246) (CST#2997), phospho-Akt1 (S473) (D9E) XP (CST #4060), phospho-Akt1 (T308) (CST #2965), Akt1 (Cell Signaling #2967), and GAPDH (Clone 6C5, Abcam).

### Immunofluorescence microscopy of AKT1-PH-EGFP fusion proteins

Wild type pcDNA3-Akt PH-EGFP plasmid was obtained from Addgene (#18836) and mutant PH domains were generated by PCR and subcloned. MCF7^PIK3CA WT^ cells were plated in an 8-well chamber slide (BD) at 2×10^4^ cells/well. The following day media was changed to serum-free media (phenol red-free DMEM/F12, 0.2% BSA), and the cells were transfected with AKT1 PH-EGFP fusion plasmids using FuGENE 6 (Promega) according to the manufacturer's instructions. Two days post-transfection, wells were washed with PBS twice, fixed in 2% paraformaldehyde (0.5 ml, 30 minutes), washed in PBS 3X, counterstained with 4',6-diamidino-2-phenylindole (DAPI, 0.3 ml/well, 0.2 mcg/ml, 20 sec), and washed once in PBS. After chamber removal, cells were mounted using Fluoromount-G, coverslipped, and sealed with clear nail polish. Images were acquired on a Nikon Eclipse confocal microscope at 60X magnification.

### Soft agar colony formation assay

3×10^4^ Rat1a cells stably expressing wild type human Akt1, Akt1 PH domain mutants, Kras G12V, or empty vector (LXSN) were seeded in 0.4% agarose in DMEM/10% FBS on top of a layer of 0.6% Ultrapure agarose (Invitrogen) in DMEM/10% FBS in duplicate in 6-well plates. Media was refreshed twice weekly, and colonies were stained with crystal violet and counted after three weeks. Bars represent mean and standard deviation.

## Supplementary Figures


