# Supplementary material for: Functional analysis of non-hotspot AKT1 mutants found in human breast cancers identifies novel driver mutations: implications for personalized medicine
Source: Oncotarget. 2012 Nov 29;4(1):29–34. doi: 10.18632/oncotarget.755 (PMC3702205; doi:10.18632/oncotarget.755)
Supplement: Supplementary file 1 [file oncotarget-04-029-s001.pdf]

Functional analysis of non-hotspot AKT1 mutants found in human breast cancers identifies novel driver mutations: implications for personalized medicine - Yi et al

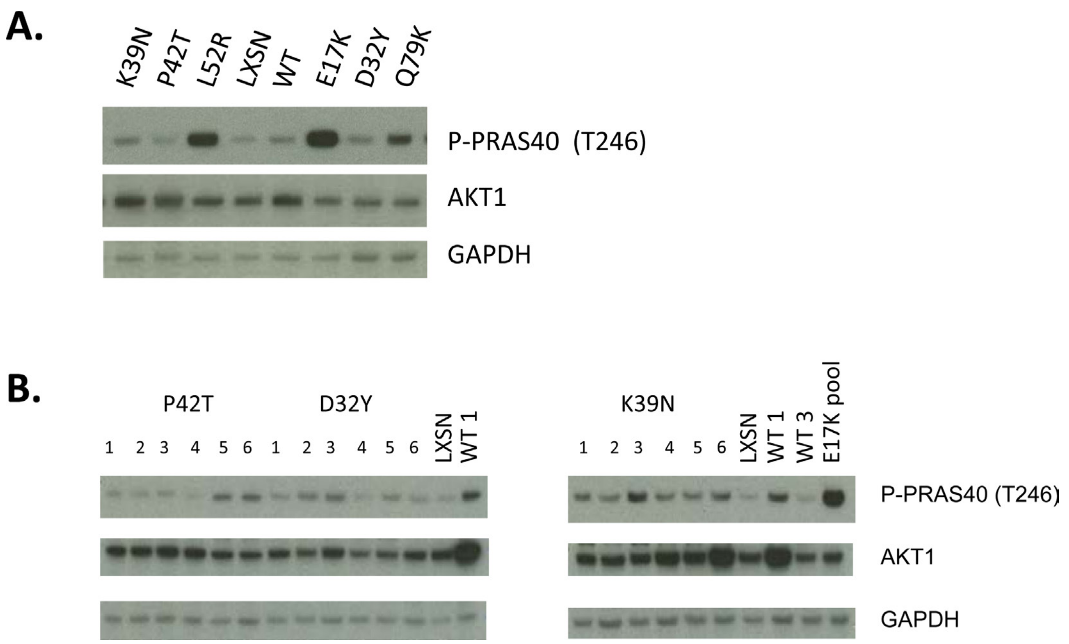

Supplemental Figure 1: The D32Y, K39N, and P42T mutants of Akt1 do not activate Akt-dependent signaling compared to wild type Akt1. A. Western blot of whole cell extracts from MCF-7^{PIK3CAWT} cells stably expressing empty vector, wild type Akt1, or Akt1 mutants (except C77F). B. Western blot of single cell clones derived from the pools of retrovirally infected MCF-7^{PIK3CAWT} cells expressing Akt1 mutants. Compared to wild type Akt1 overexpressing clones (representative low and high expressing clones are shown) and the Akt1 E17K expressing pool, the D32Y, K39N, and P42T clones do not activate Akt-dependent signaling.

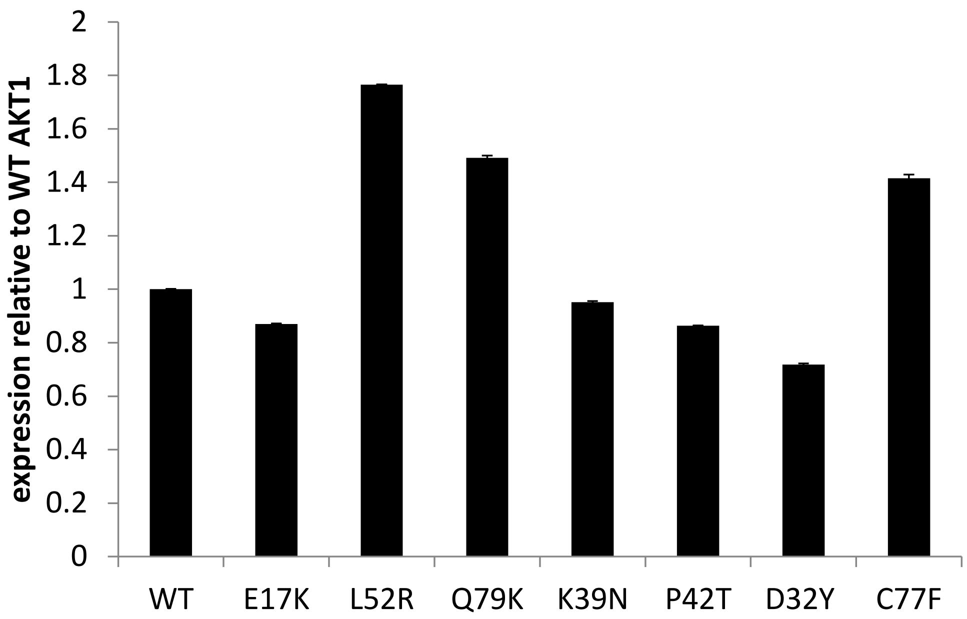

Supplemental Figure 2: Quantitative RT-PCR analysis of human *AKT1* transgene expression in Rat1a cells. Human *AKT1* mRNA levels were normalized to rat reference mRNAs. Data are depicted as expression relative to wild type (WT) human *AKT1*. Error bars depict standard deviations. Human *AKT1* transcripts were undetectable in the LXSN control cells.
